# Epidemiology of canine mammary tumours on the Canary Archipelago in Spain

**DOI:** 10.1186/s12917-022-03363-9

**Published:** 2022-07-11

**Authors:** José Rodríguez, Ángelo Santana, Pedro Herráez, David R. Killick, Antonio Espinosa de los Monteros

**Affiliations:** 1 Institute for Animal Health and Food Safety, University of Las Palmas de Gran Canaria, Las Palmas, Canary Archipelago Spain; 2 Mathematics Department, University of Las Palmas de Gran Canaria, Las Palmas, Canary Archipelago Spain; 3 Institute of Infection, Veterinary Science and Ecology, University of Liverpool, Leahurst Campus, Chester High Road, Neston, CH64 7TE UK

**Keywords:** Canine, Mammary tumour, Female dog, Breast cancer, Pathology report, Breed, Epidemiology, Veterinary, Tumour, Cancer, Neoplasia

## Abstract

**Background:**

Mammary gland tumours are the most frequently diagnosed tumours in the female dogs but just a few studies have analysed their epidemiology. Therefore, we set out to describe the epidemiology of canine mammary cancer in the Canary Archipelago, Spain. We analysed a pathology tumour registry (PTR) and identified 7362 samples obtained from 5240 female dogs resident on the Canary Archipelago during an 18-year period (2003–2020). Using a case–control study design, we compared mammary tumour affected dogs with the Canarian canine population registry in order to elucidate the breed associations for these tumours.

**Results:**

The frequency of a diagnosis of mammary tumours relative to all tumour diagnoses in female dogs decreased during the study period from 62.7% to 48.9%. Contemporaneously, the proportion of dogs diagnosed with mammary tumours who were also neutered increased from 13.6% to 26.9%. There was a negative correlation (*R* = -0.84) between these changes. Additional findings were that: the proportion of female dogs diagnosed with multiple tumours increased by 23.5% and that the proportion of malignant tumours 89.2% diagnosed has remained stable through the period. Benign mammary tumours were diagnosed at younger ages (9.2 years old) than carcinomas (9.7 years old) and sarcomas (10.4 years old). Epithelial mammary tumours were diagnosed at younger ages in entire female dogs. Samoyed, Schnauzer, Poodle, German Pinscher and Cocker Spaniel were the breeds with the highest odds-ratios (OR) in comparison with the reference (crossbreeds) while Miniature Pinscher, American Staffordshire Terrier, English Pointer as well as some local breeds such as the Canary Warren Hound and the Majorero had the lowest ORs.

**Conclusions:**

This study provides a description of the changing epidemiology of canine mammary cancer in the Canary Archipelago over the last two decades. We found high rates of CMT with a significant predominance of malignant tumours. Exact risk factors are uncertain, but a combination of environmental, regional socioeconomic affecting human and their pets, and animal management factors are likely to play a part. Specifically, neutering was negatively associated with the proportion of epithelial mammary gland tumours and breeds native to the region were at lower risk of mammary tumours. A deeper analysis of all these factors will facilitate a deeper understanding of the epidemiology of mammary gland tumours in both the canine and the human population.

## Plain English summary

In this study, we reviewed and described tumour and population data pertaining to 7362 canine mammary tumours (CMT) diagnosed over an 18-year period (2003–2020). The tumours affected 5240 female dogs (FD) from the Canary Archipelago, Spain. We compared data regarding the population of FDs affected by CMT with the Canary FD population recorded in a dog ownership registry in order to identify associations between breed and a diagnosis of CMT.

Over the course of the study period, the proportion of all tumours comprised by CMT decreased. However, the proportion of patients affected by more than one CMT increased. Contemporaneously, the neutering rate for female dogs increased significantly.

Our findings showed that almost 9 out of 10 CMT analysed were malignant epithelial tumours (carcinomas). Within this group, three types of carcinoma (complex-type carcinoma, tubulopapillary carcinoma and carcinoma arising in mixed benign tumour) accounted for almost 90% of all malignant CMT.

FD suffering from benign CMT were younger (9.2 years) that those suffering from malignant CMT (9.7 years for carcinomas and 10.4 years for sarcomas) and when the neuter status and age were considered, entire FD developed malignant epithelial CMT at younger ages (9.5 years) than neutered FD (10.2 years).

Samoyed, Schnauzer, Poodle, German Pinscher and Cocker Spaniel were shown to be at higher risk of being diagnosed with a malignant epithelial CMT compared with crossbreed dogs. Conversely, breeds such as the Miniature Pinscher, American Staffordshire Terrier, English Pointer, and some natively derived breeds such as Majorero and Canary Warren Hound were at lower risk.

## Background

CMT are the most frequently diagnosed tumours in the FD population [1–5]. Epidemiological studies [1, 5, 6] focusing on these tumours have consistently found breeds such as Poodles and Cocker Spaniels to be at greater risk of developing a CMT than cross breeds. To date, the literature contains few epidemiological studies of CMT in dogs in Spain and none in the Canary Archipelago.

In addition to these epidemiological studies a number of others have reported patient signalment alongside tumour focussed data such as the proportion of malignant versus benign tumours, the distribution of the different histological types of tumours, and the frequency with which single versus multiples CMT lesions are diagnosed in the same patient. All these previous studies used CMT collections smaller than 3000 tumours and with study periods of less than a decade [7–11].

In this paper, we have conducted an epidemiological analysis of CMT in the Canary Archipelago in the period from 2003 to 2020. Here we provide data about patient breed, neuter status and histological tumour types diagnosed and longitudinal analysis showing changes in the proportion of CMT diagnosed over time. Using a data from a mandatory dog registration scheme we describe breed associations for CMT in this region.

To our knowledge, this would be the first veterinary cancer epidemiology study developed in the Canary Archipelago and one of the largest CMT datasets described in any paper.

## Results

### Description of study populations

Two data sets were used for this study:

- The tumour dataset comprised of 7362 CMT affecting 5240 FD diagnosed from 2003 to 2020 was derived from the records of the Anatomical Pathology Diagnostic Service (APDS) of the Faculty of Veterinary Sciences of the University of Las Palmas de Gran Canaria (ULPGC), a recognized centre by the European College of Veterinary Pathologists [12]. Of the 5240 reports, 3891 (74.3%) FD presented a single CMT, while 869 (16.6%), 298 (5.7%) and 115 (2.2%) were diagnosed with 2, 3 and 4 different CMTs respectively. In 67 (1.3%) FD, 5 or more multiple mammary neoplastic nodules were identified. Table 1 shows the proportion of FD diagnosed with only benign or malignant histological types, both with single and multiple tumours, as well as FD simultaneously diagnosed with benign and malignant histological types.
  Concerning the different histological types, Tables 2 and 4 show the proportion of malignant and benign histological types respectively diagnosed between 2003 and 2010 when the former CMT classification was used [13] while Tables 3 and 5 display the proportions of malignant and benign histological types diagnosed from 2011 to 2020 on when the current CMT classification [14] was published.
  Tables 2 and 3 demonstrate that complex carcinoma, tubulopapillary carcinoma and carcinoma in benign tumour\carcinoma arising in a benign mixed tumour were the most commonly diagnosed malignant CMT over the whole study period with most of proportions being stable.
  Concerning benign histological types, simple adenoma, benign mixed tumour and complex adenoma were the most usually reported as shown in Tables 4 and 5. Most of proportions kept sable during the study period.
- The case–control dataset, consists of a subset of 1852 FD (used as cases) born between 2003 and 2013 selected from the 5240 FD included on the entire tumour dataset and of a subset of 79,100 FD born on the same period (2003–2013) obtained from a reference population (used as controls) derived from the Canary registry of animal identification (ZOOCAN). ZOOCAN is a centralized web-based registry through which veterinary practitioners from the whole Canary Archipelago have been required to register all companion animals under their care since 2011 [15].

**Table 1:** Proportion of pathology reports containing single and multiple diagnosis with benign, malignant or a mix of both histological types

Reports by CMT nodules		Proportion
Multiple Benign	50	0.95%
Multiple Malignant	1020	19.47%
Multiple Mixed	279	5.32%
Single Benign	348	6.64%
Single Malignant	3543	67.61%

**Table 2:** Proportions of the different histological types of malignant CMT diagnosed between 2003 and 2010

Histological type	Proportion (CI95%)	Trend test *p*-value*
Complex carcinoma	42.30% (40.52%; 44.10%)	0.2150
Tubulopapillary carcinoma	29.74% (28.10%; 31.42%)	0.9030
Carcinoma in benign tumour	15.70% (14.40%; 17.05%)	0.3190
Solid carcinoma	5.42% (4.64%; 6.30%)	0.8990
Carcinosarcoma	2.22% (1.72%; 2.82%)	0.4360
Carcinoma NOS^a^	1.01% (0.68%; 1.44%)	0.2250
Anaplastic carcinoma	0.94% (0.63%; 1.36%)	0.3230
In situ carcinoma	0.94% (0.63%; 1.36%)	< 0.0001
Squamous cell carcinoma	0.74% (0.46%; 1.12%)	0.0133
Osteosarcoma	0.37% (0.19%; 0.66%)	0.5560
Fibrosarcoma	0.20% (0.07%; 0.44%)	0.0879
Haemangiosarcoma	0.17% (0.05%; 0.39%)	0.4260
Lipid-rich carcinoma	0.13% (0.04%; 0.34%)	0.7220
Cribriform carcinoma	0.03% (0.00%; 0.19%)	0.4760
Osteochondrosarcoma	0.03% (0.00%; 0.19%)	0.1300
Spindle cell carcinoma	0.03% (0.00%; 0.19%)	0.7890

^a^ Carcinoma NOS refers to those diagnoses where the histological type of carcinoma has not been indicated

^*^A significant *p*-value (less than 0.05) implies some kind of trend (upward or downward) on the relative proportion of the histological type over the study period. Otherwise, the relative proportion has remained stable

**Table 3:** Proportions of the different histological types of malignant CMT diagnosed between 2011 and 2020

Histological type	Proportion (CI95%)	Trend test *p*-value*
Complex carcinoma	40.16% (38.55%; 41.78%)	0.0014
Tubulopapillary carcinoma	24.72% (23.31%; 26.16%)	0.0131
Carcinoma arising in mixed benign tumour	20.80% (19.48%; 22.16%)	0.0044
Solid carcinoma	6.86% (6.06%; 7.73%)	0.0084
Carcinosarcoma	1.89% (1.47%; 2.39%)	0.0502
Anaplastic carcinoma	1.19% (0.87%; 1.61%)	0.1250
Squamous cell carcinoma	1.11% (0.79%; 1.51%)	0.4040
Carcinoma NOS^a^	0.61% (0.38%; 0.92%)	0.1450
Osteosarcoma	0.56% (0.34%; 0.86%)	0.4110
Fibrosarcoma	0.42% (0.23%; 0.69%)	0.0477
Ductal carcinoma	0.39% (0.21%; 0.65%)	0.0019
Haemangiosarcoma	0.39% (0.21%; 0.65%)	0.2370
Sarcoma NOS^b^	0.19% (0.08%; 0.40%)	0.0446
In situ carcinoma	0.17% (0.06%; 0.36%)	0.1560
Inflammatory carcinoma	0.17% (0.06%; 0.36%)	0.4150
Carcinoma and malignant myoepithelioma	0.11% (0.03%; 0.28%)	0.6620
Malignant myoepithelioma	0.11% (0.03%; 0.28%)	0.1430
Lipid-rich (secretory) carcinoma	0.08% (0.02%; 0.24%)	0.1610
Intraductal papillary carcinoma	0.06% (0.01%; 0.20%)	0.9460
Micropapillary invasive carcinoma	0.03% (0.00%; 0.15%)	0.8270

^a^ Carcinoma NOS refers to those diagnoses where the histological type of carcinoma has not been indicated

^b^ Sarcoma NOS refers to those diagnoses where the histological type of sarcoma has not been indicated

^*^A significant *p*-value (less than 0.05) implies some kind of trend (upward or downward) on the relative proportion of the histological type over the study period. Otherwise, the relative proportion has remained stable

**Table 4:** Proportions of the different histological types of benign CMT diagnosed between 2003 and 2010

Histological type	Proportion (CI95%)	Trend test *p*-value*
Simple adenoma	38.73% (33.32%; 44.35%)	0.9160
Benign mixed tumour	34.29% (29.05%; 39.82%)	0.1550
Complex adenoma	19.05% (14.86%; 23.83%)	0.2680
Duct papilloma	2.86% (1.31%; 5.35%)	0.0025
Fibroadenoma	2.86% (1.31%; 5.35%)	0.2770
Basaloid adenoma	1.90% (0.70%; 4.10%)	0.6720
Osteoma	0.32% (0.01%; 1.76%)	0.1590

^*^A significant *p*-value (less than 0.05) implies some kind of trend (upward or downward) on the relative proportion of the histological type over the study period. Otherwise, the relative proportion has remained stable

**Table 5:** Proportions of the different histological types of benign CMT diagnosed between 2011 and 2020

Histological type	Proportion (CI95%)	Trend test *p*-value*
Simple adenoma	33.54% (29.31%; 37.98%)	0.6770
Benign mixed tumour	31.66% (27.50%; 36.04%)	0.2350
Complex adenoma (adenomyoepithelioma)	28.51% (24.50%; 32.79%)	0.4350
Fibroadenoma	2.31% (1.16%; 4.09%)	0.2860
Myoepithelioma	2.31% (1.16%; 4.09%)	0.5920
Ductal adenoma (basaloid adenoma)	1.47% (0.59%; 3.00%)	0.0235
Fibrolipoma	0.21% (0.01%; 1.16%)	0.9990

^*^A significant *p*-value (less than 0.05) implies some kind of trend (upward or downward) on the relative proportion of the histological type over the study period. Otherwise, the relative proportion has remained stable

**Fig. 1:**
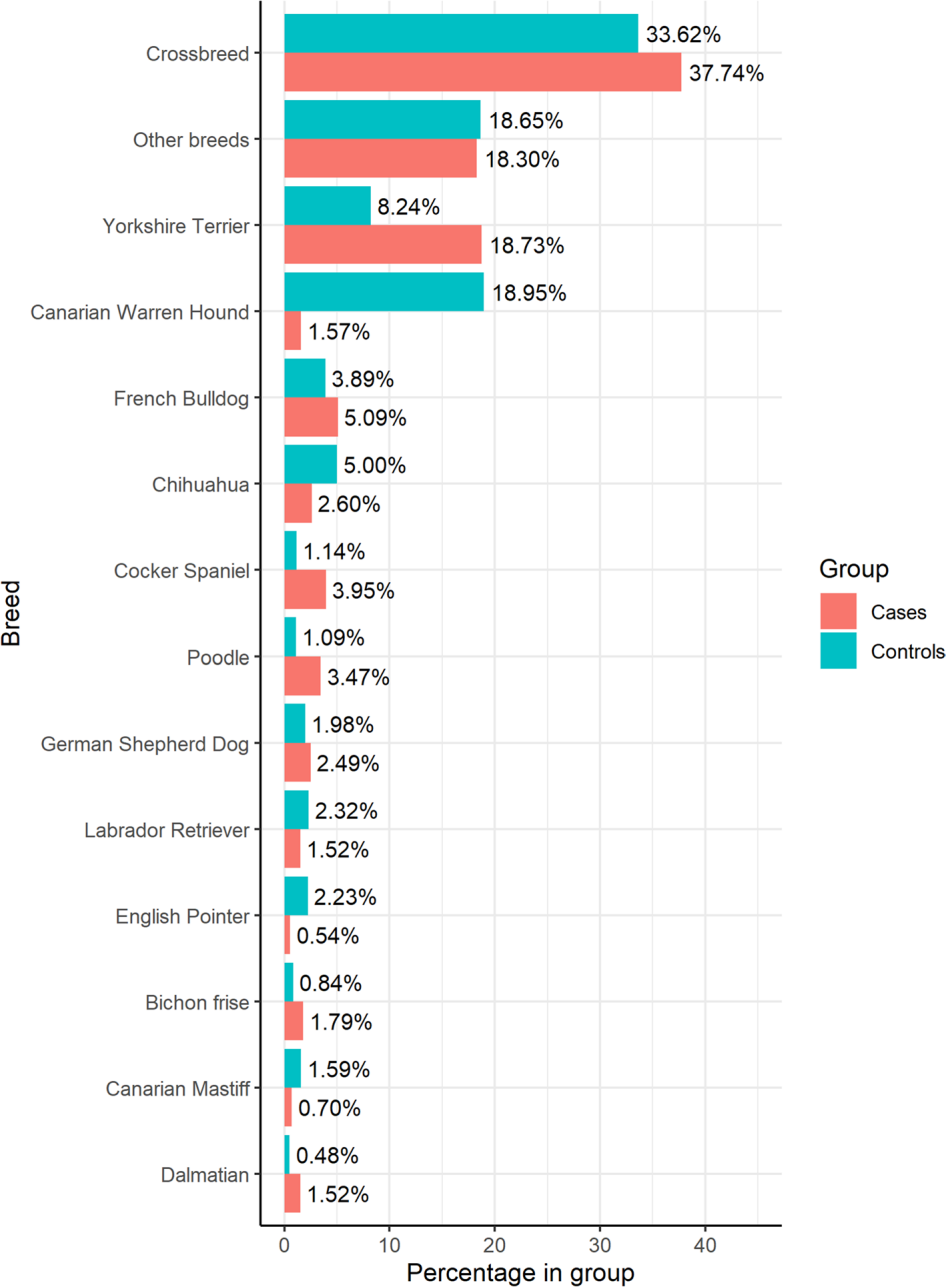
Breed distribution in cases and controls

Table 6 shows the distribution of individuals of each group (cases and controls) by year of birth while Fig. 1 shows the respective distribution of breeds.

**Table 6:** Number of cases and controls by the year of birth

Year of birth	Control	Case
2003	271	215
2004	354	221
2005	601	194
2006	945	235
2007	1470	246
2008	2274	215
2009	4182	173
2010	12,312	151
2011	19,064	94
2012	18,667	81
2013	18,960	27
Total	79,100	1852

Amongst the case group, 37.7% of dogs were crossbreed followed by Yorkshire Terriers (18.7%), other breeds group (18.3%), French Bulldogs, (5.1%), Cocker Spaniel (3.9%), Poodles (3.5%) and German Shepherd (2.5%). Within the control group, crossbreed dogs were also the largest breed group (33.6%) followed by the Canary Warren Hound (18.9%), the other breeds group (18.6%), Yorkshire Terrier (8.2%), Chihuahua (5.0%), French Bulldog (3.9%) and Labrador Retriever (2.3%).

### The CMT tumour database. An overview

**Fig. 2:**
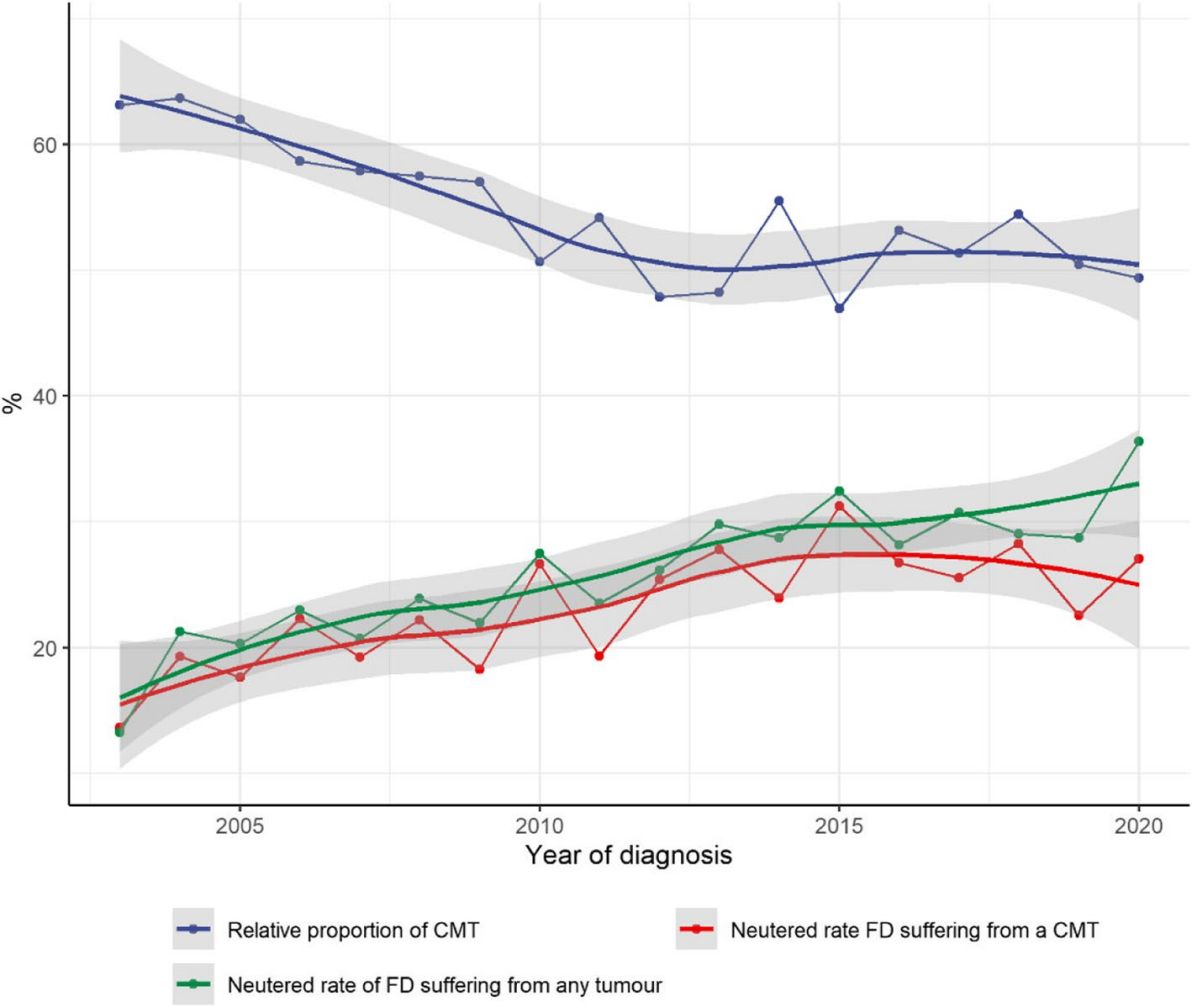
Relative proportion of CMT and neutered rate evolution in FD

During the 2003–2020 period, the APDS diagnosed 13,816 tumours from 10,205 FD from the Canary Archipelago. A longitudinal assessment showed that the proportion of CMT diagnosed dropped from 62.7% in 2003 to 48.9% in 2020 (a decrease of 13.8% (95% CI 8.4–19.0%, *p* < 0.0001)). Due to this decline CMT was no longer the most frequent tumour diagnosis at the end of the study period. Contemporaneous with this, the neutered rate of FD suffering from any tumour tripled from 13.1% to 36.3% (95% CI 17.7%-28.4%, *p* < 0.0001) showing a marked negative correlation (Pearson’s product-moment correlation: -0.84, 95% CI: -0.94 -0.60, *p* < 0.0001). Equally, the neutered rate of FD suffering from a CMT also increased significantly from 13.6% to 26.9% (95% CI 6.2%-20.6%, *p* < 0.0002) with a negative correlation of -0.84, (95% CI: -0.94 -0.61, *p* < 0.0001). These three tendencies are shown in Fig. 2.

**Fig. 3:**
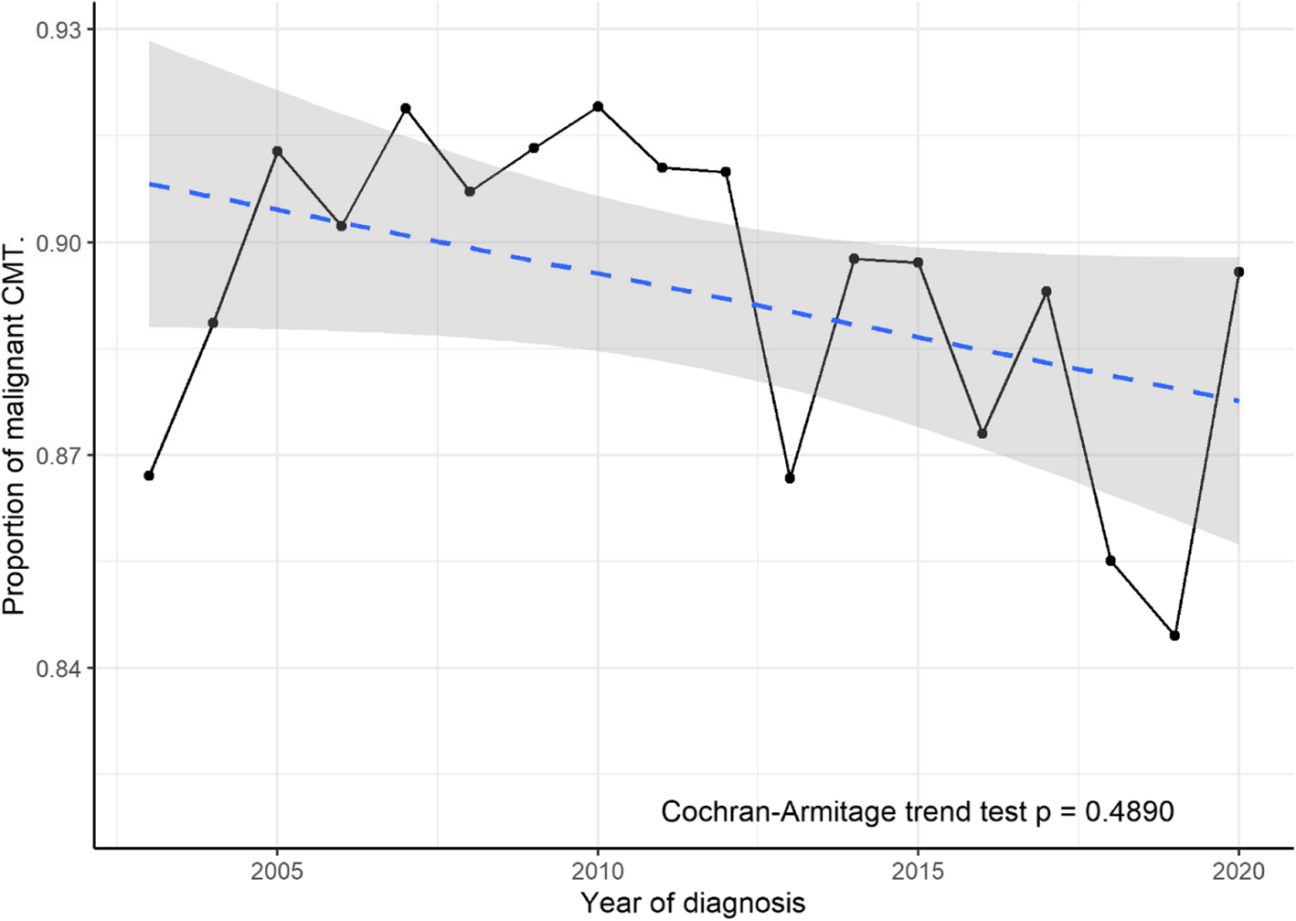
Relative proportion of malignant CMT diagnosed by year

Over the course of the study period 89.2% of the 7362 CMTs diagnosed were classified as malignant (95% CI 88.5%-89.9%). This proportion remained broadly stable across the study period as shown in Fig. 3.

### Single and multiple CMT over the study period

**Fig. 4:**
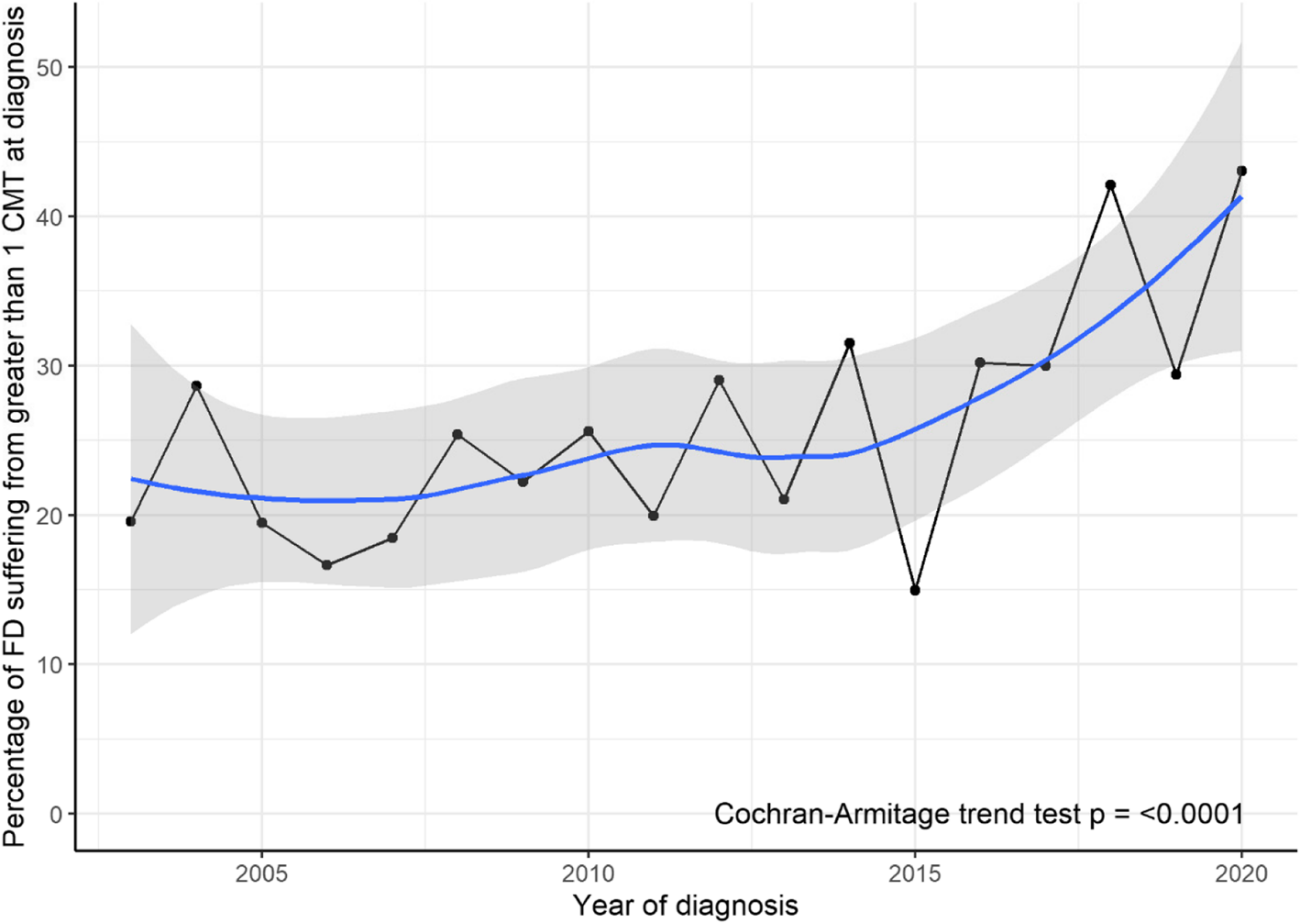
Proportion of FD with multiple CMT

Through the study period there has been an increase in the proportion of patients suffering from multiple CMT from 19.6% in 2003 to 43.0% in 2020,  an overall increase of 23.5% (95% CI 15.4–31.6, *p* < 0.0001) as shown in Fig. 4.

### Analysis of the age on the FD population affected by a CMT and its relationship with the neuter status and the presence of single and multiple tumours

The age of diagnosis in FD was analysed in comparison with the presence of single and multiple CMT, the histological type of CMT and the neuter status.

Table 7 shows the mean (± sd)) ages at diagnosis of FD depending on whether it is affected by single benign (SB), single malignant (SM), multiple benign (MB), multiple malignant (MM) or a combination of, at least, one benign CMT and one malignant CMT (MMB).

**Table 7:** Comparison of age at diagnosis by the presence of single and multiple CMT with benign, malignant or a mix of both histological types

Reports by CMT nodules	Age (mean ± sd)
Single Benign	8.57 ± 2.59
Multiple Benign	9.24 ± 2.45
Single Malignant	9.36 ± 2.63
Multiple Mixed	9.90 ± 2.23
Multiple Malignant	10.13 ± 2.32

Significant differences were found between FD suffering SB CMT compared to FD suffering from either SM CMT (*p* < 0.0001), MM CMT (*p* < 0.0001) or MMB CMT (*p* < 0.0001) as well as between FD with SM CMT and FD with either MM CMT (*p* < 0.0001) or MMB CMT (*p* = 0.012).

**Fig. 5:**
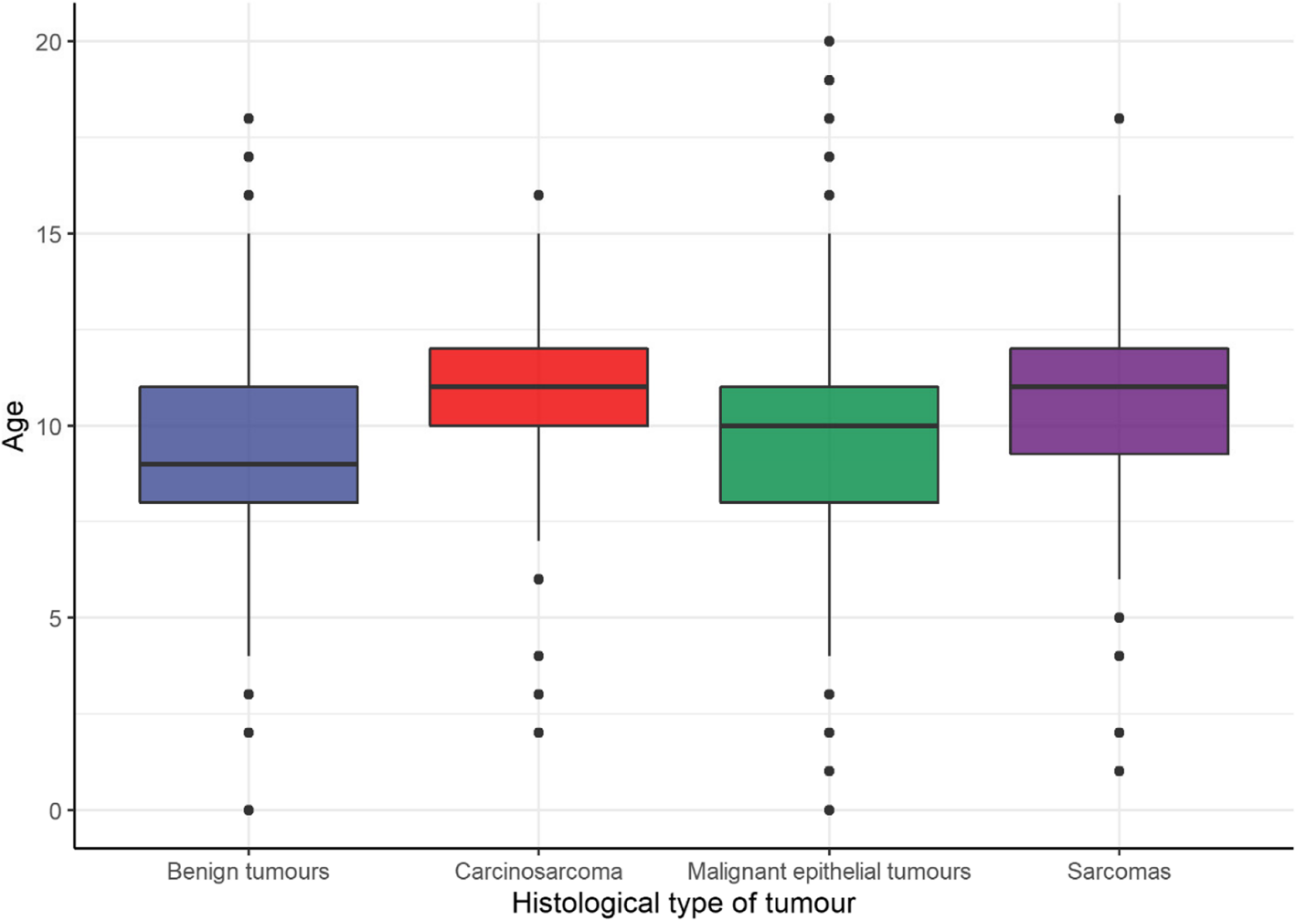
Age distribution according to the histological type of CMT

Considering the mean age at which different histological types were diagnosed, benign CMT were diagnosed at 9.2 years (sd: ± 2.57) whereas the mean age of FDs at diagnosis of carcinoma, sarcoma and carcinosarcomas was 9.7 years (sd: ± 2.50), 10.4 years (sd: ± 2.96) and 10.9 (sd: ± 2.53) respectively (Fig. 5).

Finally, neutered FD diagnosed with malignant epithelial tumours were significantly older than entire FDs diagnosed with these tumours (10.2 and 9.5 years respectively, 95% CI -0.8,-0.5, *p* < 0.0001). A similar finding was noted for benign CMT (9.7 and 8.9 years respectively, 95% CI -1.3,-0.3, *p* = 0.0005), but neither for sarcoma nor for carcinosarcoma, where *p*-value were not significant (0.9445 and 0.1921) (Table 8).

**Table 8:** Ages of diagnoses of the different CMT in both neutered and entire FD

Histological type	Difference	CI95%	Entire mean age	Neutered mean age	*P*-value
Benign tumours	-0.7830332	(-1.28,-0.29)	8.949290	9.732323	0.0005
Carcinosarcoma	-0.8279570	(-2.03,0.38)	10.688172	11.516129	0.1921
Malignant epithelial tumours	-0.6577450	(-0.85,-0.47)	9.553555	10.211300	< 0.0001
Sarcomas	0.0500000	(-1.72,1.82)	10.400000	10.350000	0.9445

### Breed as a risk factor for malignant epithelial mammary tumours

**Fig. 6:**
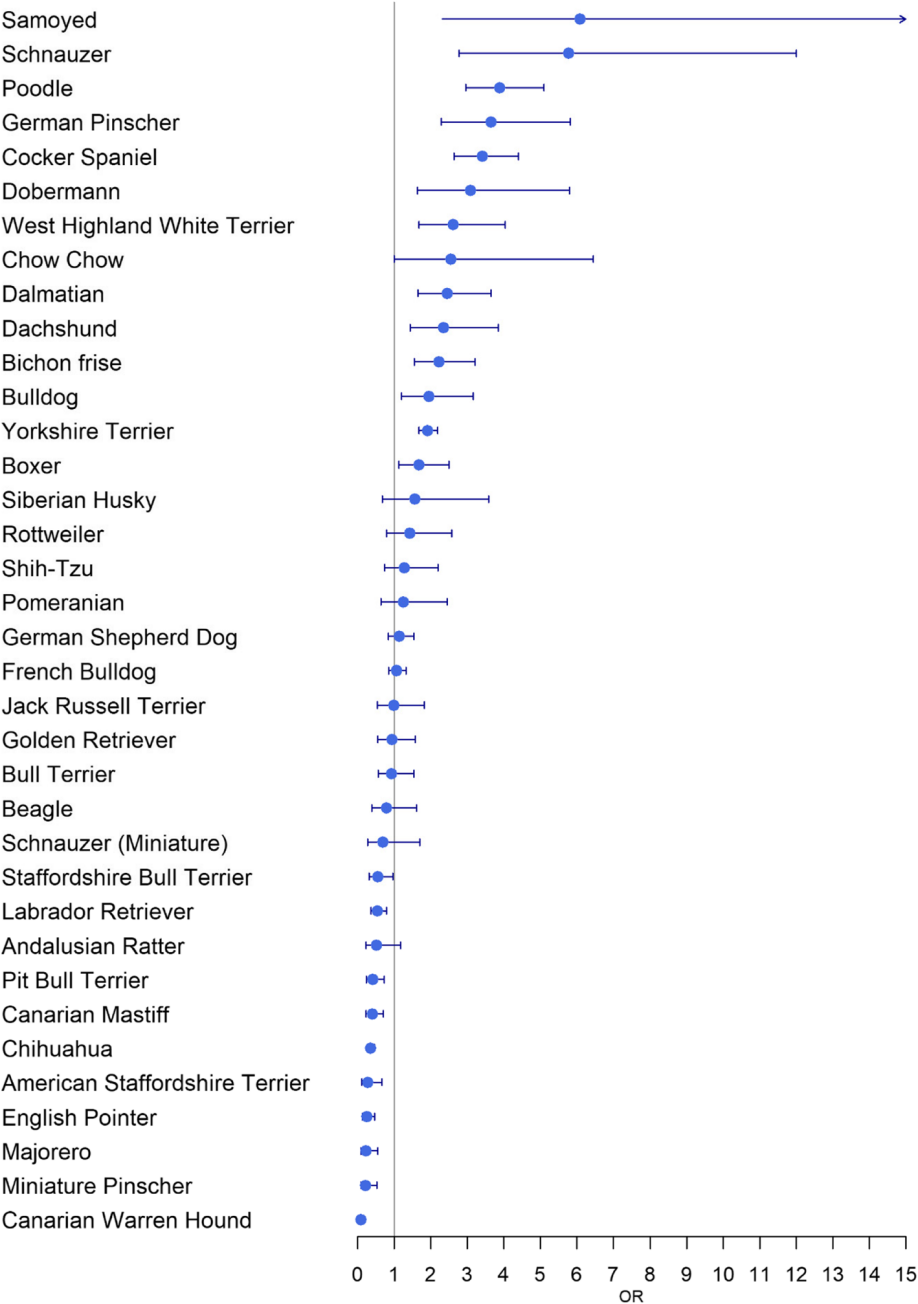
OR among different breeds when compared with crossbreed dogs

Fourteen breeds carried a significant higher risk of malignant epithelial CMT than crossbreeds. The five breeds with the highest odds ratios were Samoyed (OR 6.09, 95% CI 2.31–16.04), Schnauzer (OR 5.77, 95% CI 2.78–12.00), Poodle (OR 3.89, 95% CI 2.96–5.10), German Pinscher (OR 3.65, 95% CI 2.28–5.83) and Cocker Spaniel (OR 3.41, 95% CI 2.64–4.40). Ten breeds had a significant lower risk of malignant epithelial CMT than crossbreed, of which Canary Warren Hound (OR 0.09, 95% CI 0.06–0.13), Miniature Pinscher (OR 0.22, 95% CI 0.09–0.53), Majorero (OR 0.23, 95% CI 0.09–0.55), American English Pointer (OR 0.25, 95% CI 0.13–0.47) and American Staffordshire Terrier (OR 0.28, 95% CI 0.11–0.67) obtained the lowest OR. Table 9 and Fig. 6 show the OR by different breed.

**Table 9:** OR among different breeds when compared with crossbreed dogs, adjusted by island

Effect	Value	OR	CI	*P*-value	Controls No. (%)	Cases No. (%)
Island	Gran Canaria	1.00			25,562 (34.4)	1229 (68.7)
	Fuerteventura	0.64	[0.54,0.75]	< 0.0001	5754 (7.7)	170 (9.5)
	La Gomera	0.29	[0.15,0.56]	0.0003	706 (1.0)	9 (0.5)
	Tenerife	0.21	[0.19,0.24]	< 0.0001	31,306 (42.1)	318 (17.8)
	Lanzarote	0.20	[0.15,0.26]	< 0.0001	5694 (7.7)	55 (3.1)
	El Hierro	0.17	[0.06,0.54]	0.0026	612 (0.8)	3 (0.2)
	La Palma	0.03	[0.01,0.08]	< 0.0001	4668 (6.3)	5 (0.3)
Breed	Crossbreed	1.00			26,549 (35.7)	697 (39.0)
	Samoyed	6.09	[2.31,16.04]	0.0003	38 (0.1)	5 (0.3)
	Schnauzer	5.77	[2.78,12.00]	< 0.0001	53 (0.1)	9 (0.5)
	Poodle	3.89	[2.96,5.10]	< 0.0001	862 (1.2)	64 (3.6)
	German Pinscher	3.65	[2.28,5.83]	< 0.0001	158 (0.2)	21 (1.2)
	Cocker Spaniel	3.41	[2.64,4.40]	< 0.0001	898 (1.2)	73 (4.1)
	Dobermann	3.09	[1.64,5.80]	0.0005	143 (0.2)	11 (0.6)
	West Highland White Terrier	2.61	[1.68,4.04]	< 0.0001	302 (0.4)	23 (1.3)
	Chow Chow	2.55	[1.01,6.44]	0.0478	67 (0.1)	5 (0.3)
	Dalmatian	2.45	[1.65,3.65]	< 0.0001	382 (0.5)	28 (1.6)
	Dachshund	2.36	[1.44,3.85]	0.0006	282 (0.4)	18 (1.0)
	Bichon frise	2.23	[1.55,3.21]	< 0.0001	663 (0.9)	33 (1.8)
	Bulldog	1.95	[1.20,3.17]	0.0071	358 (0.5)	18 (1.0)
	Yorkshire Terrier	1.92	[1.68,2.19]	< 0.0001	6505 (8.8)	346 (19.3)
	Boxer	1.68	[1.13,2.50]	0.0101	656 (0.9)	27 (1.5)
	Siberian Husky	1.56	[0.68,3.58]	0.2895	153 (0.2)	6 (0.3)
	Rottweiler	1.43	[0.80,2.57]	0.2305	330 (0.4)	12 (0.7)
	Shih-Tzu	1.28	[0.75,2.21]	0.3683	434 (0.6)	14 (0.8)
	Pomeranian	1.25	[0.64,2.45]	0.5172	281 (0.4)	9 (0.5)
	German Shepherd Dog	1.14	[0.84,1.54]	0.4053	1560 (2.1)	46 (2.6)
	French Bulldog	1.07	[0.86,1.33]	0.5534	3071 (4.1)	94 (5.3)
	Jack Russell Terrier	1.00	[0.54,1.83]	0.9903	417 (0.6)	11 (0.6)
	Golden Retriever	0.94	[0.56,1.58]	0.8128	737 (1.0)	15 (0.8)
	Bull Terrier	0.93	[0.56,1.54]	0.7803	547 (0.7)	16 (0.9)
	Beagle	0.79	[0.39,1.61]	0.5253	376 (0.5)	8 (0.4)
	Schnauzer (Miniature)	0.70	[0.29,1.70]	0.4265	264 (0.4)	5 (0.3)
	Staffordshire Bull Terrier	0.55	[0.32,0.96]	0.0369	777 (1.0)	13 (0.7)
	Labrador Retriever	0.54	[0.37,0.80]	0.0017	1831 (2.5)	28 (1.6)
	Andalusian Ratter	0.52	[0.23,1.17]	0.1157	419 (0.6)	6 (0.3)
	Pit Bull Terrier	0.42	[0.24,0.73]	0.0023	1126 (1.5)	13 (0.7)
	Canarian Mastiff	0.40	[0.23,0.70]	0.0013	1253 (1.7)	13 (0.7)
	Chihuahua	0.35	[0.26,0.47]	< 0.0001	3946 (5.3)	48 (2.7)
	American Staffordshire Terrier	0.28	[0.11,0.67]	0.0043	628 (0.8)	5 (0.3)
	English Pointer	0.25	[0.13,0.47]	< 0.0001	1760 (2.4)	10 (0.6)
	Majorero	0.23	[0.09,0.55]	0.0011	615 (0.8)	5 (0.3)
	Miniature Pinscher	0.22	[0.09,0.53]	0.0008	895 (1.2)	5 (0.3)
	Canarian Warren Hound	0.09	[0.06,0.13]	< 0.0001	14,966 (20.1)	29 (1.6)

## Discussion

The aim of this study was to describe the epidemiological characteristics of CMT in the Canary Archipelago. To do this a sample of 7362 CMT diagnosed in 5240 female dogs between 2003–2020 was evaluated. Additionally, used the ZOOCAN population registry to explore the association between breed and a diagnosis of a malignant epithelial CMT within the Canarian dog population.

Over the study period, a diagnosis of CMT has become relatively less common in such a way that, at the end of the study period, tumours affecting the mammary glands were no longer the most frequently diagnosed cancer in FD. A similar downward tendency on the incidence for CMT in FD was observed in the late periods of the Animal Tumour Registry of Genoa [2].

Given the well-established protective effect of neutering on the development of CMT [16] a likely explanation for this change is an increase in the neutered rate within the FD population over the course of this period. Results of previous studies analysing animal tumour datasets and neutering rates are consistent with this hypothesis. In the Norwegian Canine Cancer Project [1], CMT were the most frequently (4223 CMT of a total of 9543) diagnosed tumours in the FD population which was described as sexually intact. On the contrary, when analysing data from a recent tumour database in the UK [17], the mammary gland was the location for just 22.5% of the tumours affecting the FD population with a neutering rate within this population of 66.4%. Additional factors that may play a part in the high rate of CMT in the Canary Archipelago include exposure to chronic and relatively high levels of contamination by Organochlorine pesticides (OCP) such as DDT and DDE found in the general population on the region given, they have been linked to breast cancer due to their role as environmental xenoestrogens [18, 19].

We found that complex carcinoma, tubulopapillary carcinoma and carcinoma arising in BMT were the most frequently ones diagnosed as has been the case in previous studies [7–11]. Despite the decrease in the diagnosis of CMT we found that the proportion of CMT that were malignant remained stable at a high level throughout the study period. The literature is somewhat discordant about the frequency of malignant CMT. Several publications found a malignancy rate of 40%-60% [6–10] conversely the Norwegian cancer register [1] and another recent study from Spain [11] found a malignant CMT rate of around 90%. Whether these results represent an inherently great likelihood that a CMT is malignant in the Canaries or is a consequence of another factor cannot be determined from this study design. In line with findings from former studies [20, 21] suggesting a progression of CMT from a benign to a more malignant phenotype, one possibility is that FDs are presented for evaluation of mammary tumour later in the disease course in the Canaries than in some other countries. Another possibility is that this finding reflects regional difference in veterinary approach leading to a submission bias due to veterinary surgeons only submitting the more phenotypically concerning tumours as well as some degree of bias inherent to Pathologists.

Consistent with the previous literature we found that CMT was usually diagnosed in older FDs and that FDs with malignant CMT were on average older at diagnosis compared to those with benign CMT [6, 9, 10, 22, 23]. Interestingly we found that epithelial CMT was diagnosed at younger ages in entire dogs than in neutered ones. This is consistent with another study [6] and provides further evidence to the protective effect of neutering in relation to CMT. Age at neutering has been shown to impact on the strength of the protective effect of neutering on CMT development [16]. Another question is whether later neutering whilst not being as protective does lead to later CMT development. As we did not have access to age at neutering data, we could not determine this from this study design.

Concerning the frequency of FD suffering multiple CMT simultaneously, the literature is somewhat inconsistent with some studies [22] reporting a rate of around 25% while others [20] demonstrated a higher one of 66.7%. In our population, the proportion of FD affected by multiple tumours increased quite significantly throughout the study period. This upward trend may be attributable to a change in approach from veterinary practitioners due to greater awareness of the so-called hormonal field effect and histological continuum from benign to malignant [20] leading to more thorough examination and earlier intervention when assessing an FD already affected by a CMT.

In this study we found higher risk breed for malignant epithelial CMT included the Samoyed, Schnauzer, Poodle, German Pinscher, Cocker Spaniel, Dobermann, West Highland White Terrier, Dalmatian, Dachshund, Yorkshire Terrier and Boxer. Lower risk breeds included the Chihuahua, English Pointer, and Labrador Retriever as well as several local breeds such as the Canarian Warren Hound, Majorero and Canarian Mastiff. Several of these breeds including Poodle, Dachshund, Cocker Spaniel have also been found to be at greater risk in studies in Norway, the US, and Italy [1, 5, 6]. Similarly Chihuahua and Pointers have been found to be at lower risk in one or more of these studies. The finding that local breeds are at lower risk is interesting especially given their varied phenotype. Further investigation is indicated to determine if this relates to some unknown environmental adaptation or possibly that for some reason they are less likely to be presented for veterinary care.

Concerning the lower ORs obtained by the different Islands in comparison to Gran Canaria, this should be attributed to a selection bias on samples from Gran Canaria.

### Limitations

#### Uncertainty of data provided by ZOOCAN

Dogs receiving veterinary care are registered on the ZOOCAN database. Typically, dogs are registered in the system when they receive their first rabies vaccination or when they are adopted from a shelter. However, follow-up information about the dog (i.e., related to changes in the neuter status or deaths) is known not to be recorded reliably on the ZOOCAN database in many cases [24]. For this reason, the only data provided by ZOOCAN used in this study was the one related to the year of birth and the breed of the dogs.

#### Breed and secondary data

The breed data used for this study was provided by veterinary practitioners and could not be checked consequently there is a degree of uncertainty accuracy of breed identification. For this reason, in this study we indicated that a dog belonged to a specific breed when that breed was clearly indicated in the report. Any combination of breeds was considered a crossbreed.

#### Selection bias in the tumour profile

As mammary tumours are superficial to the abdominal wall they are obvious to owners and vets and consequently, alongside other tumours of other superficial structures such as lymph nodes, their frequency will be overestimated relative to internal tumours [1–5]. A second likely cause of selection bias is that veterinary surgeons may be more likely to send tumours that are considered more concerning. Additionally, there might an interaction between socioeconomic factors and severity of tumour appearance such that worrisome tumours are submitted for analysis for both richer and poorer clients and less concerning ones only for wealthier clients.

Finally, geographic and logistics reasons may have led to an over-representation of cases from the island of Gran Canaria when compared with the other Canary islands. APDS is located in the Faculty of Veterinary Sciences in Gran Canaria, about thirty minutes away from the biggest urban area of the Canary Archipelago therefore the ease of submitting to APDS is clearly higher on Gran Canaria compared to the other islands and this has undermined our options to provide an island-by-island analysis on this paper due to the lack of uniformity of the submitters situation across the Canaries.

Consequently, the tumour profile obtained from the APDS service may not complete accurately recapitulate the situation in the general population. Standing against this is the nature of APDS which is a diagnostic service integrated in an academic institution, the University of Las Palmas de Gran Canaria (ULPGC) and is an affordable not for profit service aimed at teaching veterinary pathology to students. Finally, a pathological diagnosis is somewhat subjective and there is therefore a risk of pathologist bias.

## Conclusion

This study provides the first epidemiological description of FD affected by CMT on the Canary archipelago. We identified a high frequency of CMT compared to other tumours and a high rate of malignant CMT relative to most other CMT datasets. Our results support earlier observations that the age of presentation of epithelial CMT is later in neutered animals. Some local breeds like the Canary Mastiff, Majorero and Canary Warren Hound showed a lower tendency to suffer from malignant epithelial mammary tumours when compared to the crossbreed dog suggesting a possible genetic resistance adaptation developed by these breeds. A deeper analysis of all these factors could provide major insights on the epidemiology of mammary gland in the canine population.

## Methods

### The area under study

The Canary Archipelago is one of the 17 Spanish Autonomous Communities and is comprised of an archipelago of eight islands located in the Atlantic Ocean 1600 km southwest of the Spanish mainland with a population of 2.172.944 people [25] of which 80% live on the islands of Gran Canaria and Tenerife, where the two main metropolitan areas, Las Palmas de Gran Canaria and Santa Cruz de Tenerife are located.

### Data sources

Data for conducting this study come from two main sources: The APDS and ZOOCAN database.

### The APDS

The APDS receives about 1450 animal tissue samples annually. These are submitted by veterinary practitioners and official veterinarians from the whole Canary Archipelago. Along with each sample, a submission form is filled out by the vet with information about the animal from which the sample was taken (species, bred, sex, neuter status, age and location of the lesion) and about the veterinary facility where the sample was taken and submitted from.

After arriving on the APDS, samples are processed and prepared to be checked by the attending Pathologist. A diagnosis (tumour or not; in case of a tumour, the type of tumour and the grade) for each sample is indicated on the same case-report document used to submit the sample generating a diagnostic report.

Finally, both the diagnostic report and the processed sample are stored on the ADPS archives for further review if needed. For this study, tumour-related data came from the archived diagnostic reports covering the period 2003 to 2020.

Over the study period, there have been three main Pathologists working the samples with the role of Veterinary Pathology Professors of the University of Las Palmas de Gran Canaria these have been supported by occasional residents.

### The ZOOCAN database

The ZOOCAN database is a centralized web-based registry in which veterinary practitioners from the whole Canary Archipelago must register all companion animals under their care. In the Canary Archipelago, it is mandatory to identify dogs once they are three months old [13]. Rabies vaccination is also mandatory from this age and this vaccination should be always preceded by the registration of the animal on the database. It is also possible to register dogs younger than three months of age.

The database is managed by the Regional College of Veterinary Surgeons who provided the anonymized dataset used in this study in the form of a*.csv* file containing 369,083 rows and 6 rows concerning to breed, gender, date of birth, neuter status, island of residence and date of registration.

As explained previously, a subset of this whole collection of data (female dogs born between 2003 and 2013) was selected as controls (non-cases) to shape the study population.

## Data preparation

### The tumour database

Initially, all APDS archives were in paper format so it was necessary to create a digital database (a pathology tumour registry) for its further analyses.

For this purpose, all diagnostic reports generated on the 2003–2020 period were individually read by a single author (JRT). Data from records describing a tumour in a dog or a cat was extracted and added to the registry.

Data was recorded in an MS Excel spreadsheet in a two steps process from which two subsets of data were created: a first one with only tumour related data (histological type, grade, location and whether cytology or histology) and a second one with animal data (species, breed, neuter status, age and place of living). Both subsets were merged afterwards using R by making use of the unique reference number.

At the end of the process, from 25,957 reviewed reports, 12,330 included at least a tumor lesion in a FD.

### Case-definition: gland mammary histological type subset and multiple tumour cases

The CMT subset from the whole cancer registry was obtained by firstly filtering by species and gender (canine, female), next by the tumour location (mammary gland). When selecting the control cases for comparison with the reference population, a third filter was applied by the year of birth (2003–2013).

Given the long period of time, classification systems applied for the CMT have changed over the years so, for this study, mammary gland tumours were described according to either the current [24] or the former [25] CMT classification system.

Concerning multiple tumour cases, these refer to pathology reports including more than one CMT.

### Breed name standardization

Given the high diversity of ways used to indicate the breeds of the dogs (for instance “Labrador Retriever”, “Retriever, Labrador”, “Lab”, “L. Retriever”, it was necessary to standardize all these terms by mapping to the lists of the Fédération Cynologique Internationale (FCI) and the Royal Spanish Canine Society augmented by recent additions based on popular hybrids (e.g. El Hierro Wolfdog, American Bullie).

### Study design

In a case–control study, cases (animal with a disease) and controls (animals known to be free of the disease) are selected from the population of interest in such a way that both groups have similar characteristics that make them comparable to each other. In this kind of study, it is ideal to have at least as many controls as cases, in order to improve the efficiency of statistical analysis.

In this sense, both cases and controls consisted of individuals coming from the same Autonomous Community (Canarias) presumably from dogs living in this Autonomous Community (given the geographical separation of the Canaries from mainland Spain), while animals registered in ZOOCAN are those living in any city or town of the Canary Archipelago.

However, given that CMT affect elderly female dogs, it was necessary to choose a subset of individuals from the ZOOCAN database that also met this age-related requirement.

So, to meet the requirement of having so many controls as cases individuals, we chose a subset of FD born within the 2003–2013 period in which the number of animals on the control group was higher than of the one on the case group as shown in Table 6.

### Statistical analysis

After performing an internal validity check and data cleaning with Microsoft Office Excel 2013, all analysis were performed with the R Language and Environment for Statistical Computing, version 4.1.2 [26]. Categorical variables were expressed as numbers and percentages, and continuous symmetric distributed variables (age) were expressed as the mean and standard deviation. Differences in the age at presentation for the different histological type of tumours depending on the neuter status were assessed by using t-test. The Cochran-Armitage trend test was used for assessing the presence of increasing or decreasing trend in proportions. Difference in proportions was assessed by chi-square tests and corresponding 95% confidence intervals were computed. Linear regression analysis was used to assess the annual growth rate in the proportion of various tumour types. Kruskal Wallis test was used to assess the differences between ages at diagnosis for different combinations of benign and malignant and single and multiple tumours. Additionally, when Kruskall Wallis provided significant results, a post hoc Conover test was used to find out which groups were significantly different.

Linear association between continuous variables was assessed by Pearson correlation coefficient. Logistic regression analysis was used to evaluate the association of particular breed with the risk of malignant epithelial CMT adjusted by island, and odds ratios with 95% confidence intervals were reported. Only malignant epithelial tumours (carcinomas) were evaluated given that this was the largest homogeneous histological type group. Crossbreed dogs were used as the reference group. Additionally, given the overrepresentation of cases in the island of Gran Canaria, the logistic model was adjusted by island.

In all tests, *p*-values lower than 0.05 were considered as statistically significant. The R script used to execute the above analyses is available at https://doi.org/10.6084/m9.figshare.19688721

## Data Availability

The datasets generated and/or analysed during the current study are available at https://doi.org/10.6084/m9.figshare.19688721
